# The impact of the COVID-19 pandemic on emergency department visits in region Stockholm: a population-based study between 2018-2021

**DOI:** 10.1186/s12889-026-29278-w

**Published:** 2026-09-08

**Authors:** Megan Doheny, Janne Agerholm, Bo Burström, Ann Liljas

**Affiliations:** 1 Department of Neurobiology, Care Sciences and Society, Karolinska Institutet, Aging Research Center, Tomtebodavägen 18A, plan 10, Stockholm, 171 65 Sweden; 2 Department of Global Public Health, Karolinska Institutet, Stockholm, Sweden; 3 Centre for Epidemiology and Community Medicine, Stockholm County Council, Stockholm, Sweden

**Keywords:** Emergency department visits, COVID-19 pandemic, Time series analysis, Emergency care sensitive conditions, Socio-demographic differences

## Abstract

**Background:**

As COVID-19 cases increased, organizational changes were implemented in Emergency Departments (EDs) to manage the expected demand. However, the impact of these changes on ED utilization is not understood. This study aims to examine the pattern of hospital-based all-cause ED visits and ED visits for Emergency Care Sensitive Conditions (ECSC) and external causes of morbidity (e.g., falls, traffic accidents) between 2018 and 2021 and assess the effect of the COVID-19 pandemic on ED care utilization by socio-demographic groups.

**Methods:**

A population-based study including those 18 years and older in region Stockholm (*N* = 1, 848,271) comparing the utilization of hospital-based ED care obtained from the National Patient Register between 2018 and 2021. The pattern of ED visits was visually assessed, the types of ED visits and socio-demographic composition described, and an interrupted time series analysis was used to assess trend changes between 2018 and 2021.

**Results:**

Between 2018 and 2019 the weekly volume of ED visits decreased, followed by a sharp decrease in 2020 and remaining at a reduced level in 2021. ED visits due to external causes followed a similar trend, while the rate of visits for ECSC increased between 2018 and 2019. The trend in ED visits per week decreased by -11.6 (CI: -13.8 to -9.3) between 2018w1-2020w10, a significant level change of -1,175 0.0 ED visits (CI: -1,418.5 to-932.6) compared to 2020w11. ED utilization decreased across socio-demographic groups to various extents although remained lower at the end of 2021 compared to 2018–2019.

**Conclusions:**

The reduced utilization of ED care for ECSCs and across socio-demographic groups may indicate that individuals delayed or avoided seeking necessary care during the COVID-19 pandemic. These findings are important in guiding health policy and resource allocation. Moreover, further research is needed to determine whether this decline is due to seeking alternative care sources or forgoing care altogether.

**Registration trial:**

Not applicable.

**Supplementary Information:**

The online version contains supplementary material available at https://doi.org/10.1186/s12889-026-29278-w.

## Background

The rapid rise in the number of cases and subsequent hospitalizations during the Coronavirus (COVID-19) pandemic placed enormous pressure on the Swedish healthcare system due to the restructuring of essential healthcare services, and especially the Emergency Department (ED) where the mixing of COVID-19 patients with other patients had to be limited [1–4]. Simultaneously, the government implemented policies and recommendations to limit community spread of the virus which disrupted the day-to-day lives of the population and altered their healthcare seeking behavior [3, 4].

In Sweden, there are three levels of governance: national, regional and municipal. The 21 regions are responsible for the organization and financing of health and medical care, including disease prevention and control [5]. Many Swedish government agencies across all levels were involved in the COVID-19 response in their respective jurisdictions including but not limited to travel, education, medical care and eldercare, the response included both legal regulations to regions and municipalities, as well as strong but voluntary recommendations to the population [2–4]. The Swedish Public Health Agency was responsible for coordinating the national response throughout the Covid-19 pandemic [6].

During the first wave of the COVID-19 pandemic, changes were made to how healthcare was delivered and accessed in Sweden. For example, between the 2nd and 13th of March 2020 the population was advised to always contact the regional medical advice call center (“1177”) for advice on when and where to seek care before coming in [7]. Further, the regions launched an online self-triage tool and the guidelines for seeking ED care were strict, specifying that if a patient was not experiencing rapid health deterioration, they should seek alternative care or continue self-care at home [8]. On March 20, 2020, the National Board of Health and Welfare (NBHW) introduced more stringent recommendations requiring external triage of walk-in patients at hospital-based EDs. Triage was performed in tents outside the ED by a nurse in PPE maintaining a two-meter distance, separating patients with suspected COVID-19 from others. Patients without COVID-19 symptoms were directed to the emergency room or discharged home, while suspected cases were admitted for further assessment based on severity [2].

A reduction in the number of ED visits during the first wave of the COVID-19 pandemic was observed in many countries [9–16]. For example, a 35% decrease in ED attendance was reported in London [16], and a large national American study found a 42% reduction in ED visits in April 2020 compared to 2019 [15]. A similar trend was observed in Sweden, where ED visits decreased by 31% between March-April 2020 compared to previous years [17]. While some reduction in ED visits may reflect a decrease in inappropriate or non-urgent healthcare seeking, concerns remain about unmet care needs for conditions requiring timely emergency care. A comprehensive list of Emergency Care Sensitive Conditions (ECSCs) has been compiled and consists of acute conditions and diseases that require immediate emergency care [18, 19]. Decreases in ED visits for ECSCs during the COVID-19 pandemic have been reported [20, 21], including reductions in visits related to myocardial infarction, stroke, and other cardiac complaints during the first wave [22].

Previous studies have highlighted that patients delayed seeking ED care for several reasons, such as not wanting to burden the healthcare services, concerns for nosocomial infection, and perceiving their own healthcare needs to be less acute compared to patients with COVID-19 [9, 23, 24]. Particularly older people were found to defer seeking healthcare to a greater extent than younger age groups during the COVID-19 pandemic [25]. Socio-demographic differences in ED utilization have also been observed, as a pre-COVID-19 study set in Stockholm County found that among persons 65 years and older, lower income groups and women were more likely to be frequent ED users compared to higher income groups [26].

The reconfiguration of healthcare resources to deal with the expected inflow of COVID-19 patients combined with recommendations to limit community spread raised concerns about the potential collateral damage for non-COVID-19 patients [23]. Most previous studies have focused on the initial reduction in the volume of ED visits during the first wave of the pandemic, or the impact on individual hospitals [9, 15, 16, 27]. This study aims to examine the patterns of hospital-based ED visits, ED visits for ECSCs, and visits for external causes of morbidity in Stockholm County from 2018 to 2021, and assess the effect of the COVID-19 pandemic on the utilization of ED care, stratified by socio-demographic groups.

## Methods

A population-based study using linked register data to assess the pattern of hospital-based ED care among the inhabitants of Stockholm County between 2018 and 2021. The study population was defined in the Total Population Register (*Registret över Totalbefolkning)* (RTB) which includes all registered inhabitants 18 years and older (*N* = 1,848,271) registered as residents of Stockholm Region during 2019–2020, were followed using healthcare utilization data covering the period 2018–2021. From the RTB demographic variables were measured including sex as male or female; age measured using year of birth and categorized into intervals of 18–39, 40–49, 50–59, 60–69, 70–79 and 80 + years. Country of birth was dichotomized as born in Sweden or elsewhere.

Measures of socio-economic position (SEP) were obtained from the Longitudinal Integrated Database for Social Insurance and Labor Market Studies (LISA) which is a collection of variables from several different administrative registers. Information from LISA was obtained for 2018, and SEP was measured based on level of education, which was categorized as primary, secondary, and tertiary, as well as equalized disposable household income that was ranked into quintiles from the lowest to highest based on the distribution in the population.

### Emergency department visits

This study used data retrieved from Swedish National Patient Register (NPR). The outcome all-cause ED visits were defined as any hospital-based ED visit that involved consultation with a medical doctor in Stockholm County between 2018 and 2021. These diagnoses were recorded at the end of each ED visit. For the purposes of this study, ED visits for ECSC were measured based on the main diagnosis registered at the end of an ED visit in the NPR, based on 51 conditions identified as ECSCs by Vashi et al., the full list of which, including corresponding ICD-10 codes, is available in the supplementary material of Vashi et al. [19]. These conditions affect adults across a wide age spectrum and require timely access to emergency care, as rapid identification and treatment improves patient outcomes (both morbidity and mortality) [19]. External causes of injury were defined as ICD-10 codes which classify unintentional, accidental, or intentional injuries that necessitate emergency care. These included ED visits due to: transport (motor and non-motor vehicle accidents), fall, drowning, suffocation, nature, accidental poisoning, machinery, burns, assault, self-harm, complications due to medication or surgical procedures, and unspecified [28, 29].

### Data analysis

The patterns of hospital-based ED visits (all-cause, ECSCs, external causes, and COVID-19 related) among the inhabitants of Stockholm County between 2018 and 2021 were visually assessed, by calculating the moving average for 4.1 segments for the weekly number of ED visits, to remove seasonality. The age standardized rate of ED visits and ED visits for ECSC were calculated per 1,000 persons for socio-demographic groups between 2018 and 2021 based on population weights from the European standard population [30].

The weekly number of ED visits was analyzed in an Interrupted Time Series (ITS) analysis to assess the effect of the COVID-19 pandemic on the utilization of ED care among inhabitants of Stockholm County. The interruption was defined as when the Swedish Public Health Agency elevated their risk assessment to “a high risk of spread in Sweden” which was the point when societal measures and healthcare regulations were introduced to cope with the COVID-19 pandemic and occurred on week 11 in 2020 [2]. The ITS analysis included 208 time points of which there were 114 time points before week 11 2020 and 93 time points after the interruption. The single ITS model included a time variable that counted the number of time points in the study period, which estimates the trend of the outcome before week 11 2020, a dummy variable distinguishing the time periods before and after week 11 2020, and an interaction variable between time and the dummy variable to determine the trend change in ED visits after week 11 2020. The ITS was performed using an ordinary least squares regression model with Newey-West standard errors, which are robust in the presence of autocorrelation, and the predicted probabilities were estimated to fit the linear trends [31, 32].

## Results

**Fig. 1:**
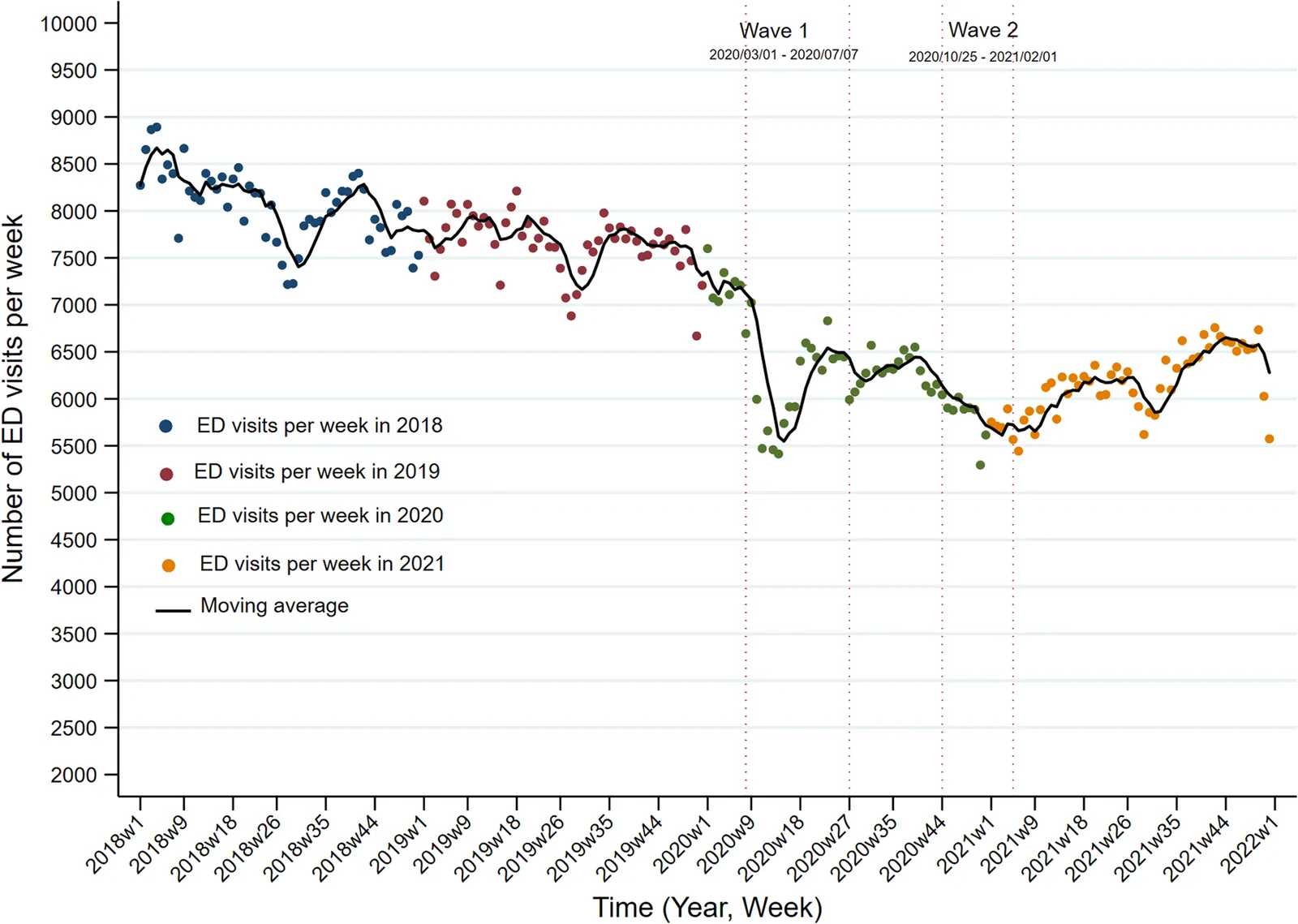
Number of Emergency Department (ED) visits per week and the 4-week moving average among inhabitants aged 18 years and older in Stockholm County between 2018–2021. Note the gradual decline in ED visits prior to 2020, which may partly reflect structural healthcare reforms in Stockholm discussed in the Discussion section

The weekly volume of hospital-based ED visits among the population of Stockholm County between 2018 and 2021 is presented in Fig. 1. There were 413,077 all-cause hospital-based ED visits in 2018 which decreased by 6.2% between 2018 and 19. This decline preceded the COVID-19 pandemic and may in part reflect ongoing structural changes in emergency care organization in Stockholm County, which are discussed further below. The number of all-cause ED visits decreased by 18.6% between 2019 and 2020 (see Table S1). The sharpest decrease occurred between week 9 in 2020 (2020w9) and week 27 (2020w27), which corresponds to the first wave of the COVID-19 pandemic, after which it increases slightly until the second wave as depicted in Fig. 1. There was a further decrease in all-cause ED visits by 3.7% between 2020 and 2021. The weekly volume of ED visits increased again towards the end of 2021, but not back to the pre-pandemic level.

**Fig. 2:**
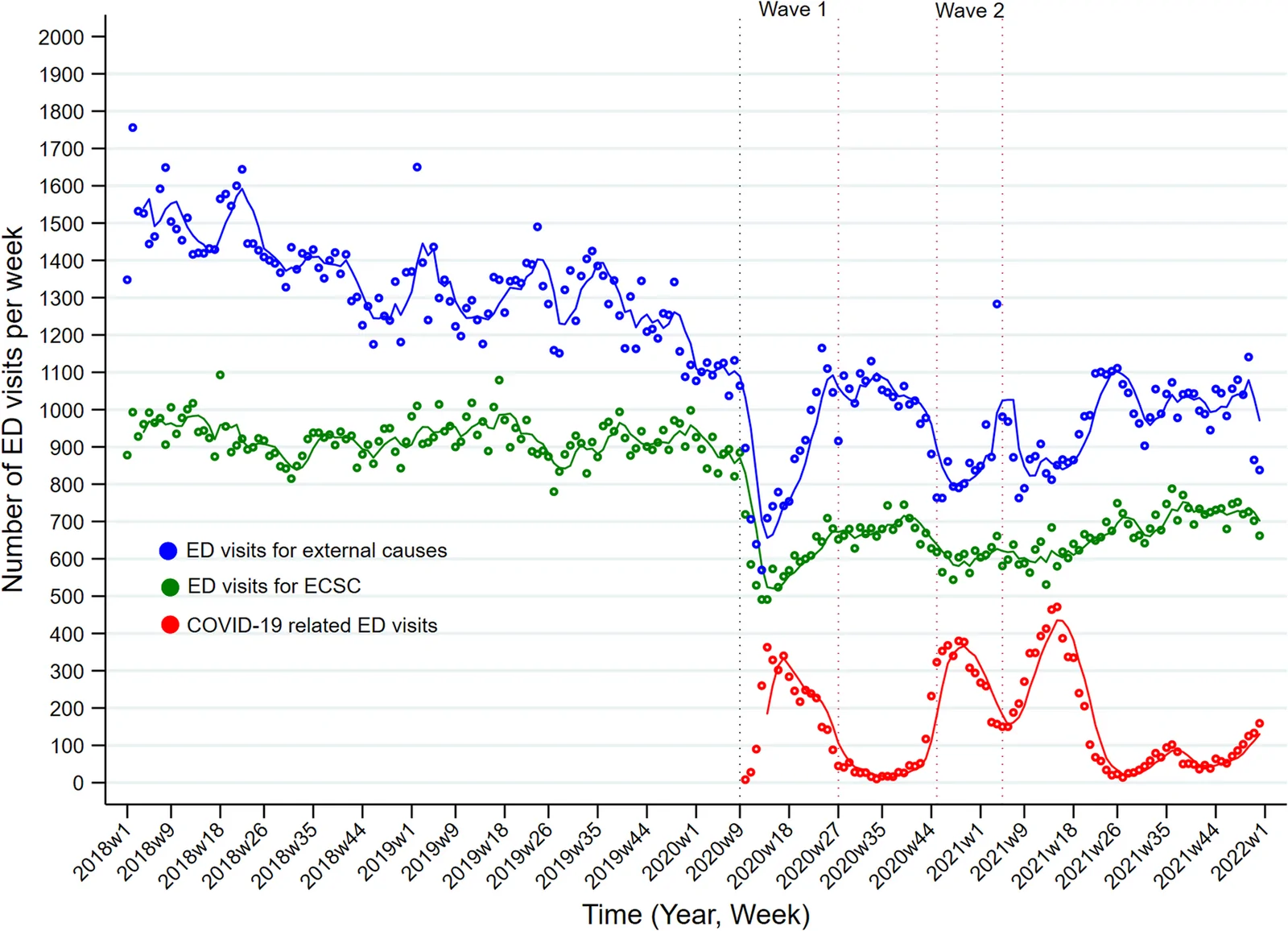
Weekly number of ED visits for Emergency Care Sensitive Conditions (ECSC), external causes of morbidity, and COVID-19, among inhabitants aged 18 years and older in Stockholm County, 2018–2021

Figure 2 depicts the weekly number of ED visits for ECSC, external causes, and visits related to COVID-19 infection between 2018 and 2021. The weekly number of ED visits for ECSCs is depicted by green circles and the moving average by the green line in Fig. 2. ED visits for ECSCs increased slightly in the period preceding the first pandemic wave, and after an initial drop, visit numbers gradually recovered after the wave subsided, though changes between 2020 and 2021 were less pronounced.

The rate of ED visits for external causes decreased between 2018 and 2019 (see Table S1) and there was a further decrease in 2020. In 2020, there were 44.7 visits per 1,000 persons, while there was a slight increase to 46.2 visits per 1,000 persons in 2021. In Fig. 2, the blue circles represent the weekly number of ED visits for external causes, and the blue line represents the moving average. The decreases in the weekly number of ED visits for external causes corresponded to increases in ED visits related to COVID-19 infection (depicted by the red line and circles in Fig. 2) during the first and second waves. The composition of ED visits due to external causes did not vary greatly between 2018 and 2021 (see Table S2), most were fall-related which varied from 46.2% in 2018 to 51.0% in 2021. In 2020, there was a slight increase in the proportion of visits due to assault and self-harm compared to 2018–2019, while in 2021 there was an increase in the proportion of ED visits for external causes due to complications related to medication or treatment.

### Socio-demographic differences in ED visits

The study population and the age standardized rates of ED visits per 1,000 persons between 2018 and 2021 is shown in Table 1. The study population was *N* = 1,848,271, out of which 50.4% were female, the mean age was 47.1 years, 29.7% were born outside of Sweden, and 44.2% and 38.7% had completed secondary and tertiary education, respectively. The age standardized rate of ED visits decreased from 186.2 visits per 1,000 persons in 2018, to 138.9 visits per 1,000 persons in 2021. Female inhabitants had a higher rate of ED visits than male inhabitants from 2018 to 2021. All-cause ED visit rates were highest among those aged 18–39, while ED visit rates for ECSCs were highest among older age groups (see Table S3). Between 2018 and 2019 the all-cause ED visit rates were decreasing among all age groups except for those 70 years and older, for whom there was a slight increase. Similarly, the rate of ED visits for ECSC increased among those 80 years and over, from 5.1 visits per 1,000 persons in 2018 to 5.7 in 2019. Consistently, there was a higher rate of ED visits among persons with secondary education, and those in the lowest income quintile between 2018 and 2021. ED visit rates decreased across all age groups in 2020. In 2021, both all-cause and ECSC-specific rates increased, though only among those aged 60–69 and 70–79.

**Table 1:** Socio-demographic characteristics of the study population and the age-standardised rates of ED visits per 1,000 persons Stockholm County, 2018–2021

	*N = 1,848,271*	186.2	175.8	143.0	138.9
Sex					
Female	50.4%	102.5	95.7	76.3	74.6
Male	49.6%	83.7	80.1	66.7	64.3
Age groups					
18-39yrs	39.9%	43.1	39.0	31.9	29.0
40-49yrs	17.8%	22.5	19.9	17.4	16.6
50-59yrs	15.9%	26.0	24.1	21.0	20.5
60-69yrs	11.8%	29.5	27.4	22.7	23.2
70-79yrs	9.7%	32.7	32.8	26.0	27.1
80 + yrs	4.9%	32.3	33.4	24.7	22.7
Country of birth					
Other	29.7%	55.0	51.0	42.6	41.3
Sweden	70.3%	131.2	124.8	100.4	97.6
Education level					
Primary	13.7%	40.0	38.0	30.4	29.1
Secondary	44.2%	85.4	79.9	65.0	63.2
Tertiary	38.7%	56.3	53.2	43.9	43.1
Missing	3.4%	4.5	4.8	3.6	3.5
Income groups					
(low) group 1	19.6%	52.8	50.0	40.3	38.2
Group 2	19.6%	45.6	43.1	34.5	33.6
Group 3	19.6%	31.7	31.7	24.4	24.1
Group 4	19.6%	27.4	25.8	21.4	21.1
(high) group 5	19.6%	27.6	25.9	21.3	21.0
Missing	2.0%	1.1	1.2	1.0	0.9

*(%) = proportion of the total population, *Age standardized rates per 1,000 persons, based on weights of the European standard population

### Interrupted times series analysis

Estimates of the trend changes in all-cause ED visits from 2018w1 to 2021w52, derived from the ITS analysis, are shown in Table 2. There was a significant decreasing trend in the weekly number of all-cause ED visits, corresponding to a decrease of -11.6 ED visits per week (95% CI: -13.8 to -9.3) from 2018w1 to 2020w10. In week 2020w11 there was a significant level change in the number of ED visits of -1,175 ED visits (CI: -1,4186.5 to -932.6) among the total population. There was also a gradually increasing trend of 13.8 all-cause ED visits per week (CI: 9.6 to 18.1) from 2020w12 to 2021w52. The predicted trend changes are shown in Figure S1.

**Table 2:** Interrupted time series estimates of changes in the weekly number of ED visits in Stockholm County, 2018w1 and 2021w52

	Coef.	CI 95%	Coef.	CI 95%	Coef.	CI 95%
*All-cause ED visits*	-11.6	(-13.8 to -9.3)	-1,175.0	(-1,418.5 to -932.6)	13.8	(9.6 to 18.1)
*Socio-demographic groups*						
70 + years	-1.1	(-2.0 to -0.2)	-614.8	(-721.3 to -508.2)	3.6	(3.2 to 1.8)
Male	-4.1	(-5.4 to -3.4)	-472.4	(-579.9 to -364.9)	4.8	(3.0 to 6.6)
Female	-7.2	(-8.6 to -5.7)	-703.2	(-851.2 to -555.1)	9.1	(6.4 to 11.7)
Born outside of Sweden	-2.6	(-3.4 to -1.8)	-340.9	(-404.0 to-277.7)	4.0	(2.8 to 5.3)
Primary level education	-2.2	(-2.6 to -1.7)	-283.2	(-333.5 to -232.9)	2.5	(1.7 to 3.4)
Lowest income group	-3.0	(-3.5 to -2.5)	-366.7	(-428.6 to -302.9)	3.3	(2.3 to 4.3)

*Coef – ITS estimates of the changes in the number of ED visits *C\I – confidence intervals 95%

Similar trend changes were observed across socio-demographic groups (Figure S2), though among those aged 70 and older, the slight pre-pandemic decreasing trend of -1.1 visits per week (CI: -2.0 to -0.2) was not significant. A significant level change in ED visits was associated with 2020w11, followed by a significantly increasing trend across all socio-demographic groups, albeit to varying extents. Despite this recovery, weekly ED visit volumes remained below pre-pandemic levels through the end of 2021.

## Discussion

This study examined changes in hospital-based ED visit patterns among inhabitants aged 18 and older in Stockholm County between 2018 and 2021. ED visit volumes declined slightly between 2018 and 2019, followed by a sharp decrease at the onset of the COVID-19 pandemic in 2020. After this initial drop, volumes fluctuated before increasing slightly toward the end of 2021, though not returning to pre-pandemic levels. Similar patterns were observed for ED visits related to external causes, with some variation in the distribution of specific causes in 2020-21 compared to 2018-19. For ECSCs, visit rates increased slightly between 2018 and 2019, with higher rates observed among females, older age groups, and those with tertiary education. A sharp decrease followed between 2019 and 2020, but differences in ECSC visit rates between 2020 and 2021 were less pronounced. Higher rates of all-cause and external cause ED visits were observed among females, older age groups, lower-income groups, and persons born outside of Sweden, while those aged 18–39 had higher rates of ED visits for external causes. Prior to the pandemic, a declining trend in weekly hospital-based ED visits was observed across all socio-demographic groups, though not statistically significant among those aged 70 and older. All groups broadly followed similar trend changes during the study period, though overall ED utilization did not recover to 2018-19 levels by the end of 2021.

The decreased utilization of hospital-based ED care observed in this study may reflect a decrease in the actual need of emergency care, a decrease in access to hospital-based ED care or a combination of both. The objective of emergency care is to stabilize and help patients through the most critical part of illnesses or injuries, making estimation of actual need of emergency care difficult. However, there was a reduction in the overall incidence rate of myocardial infarction during the first few weeks of the pandemic with 21% fewer cases reported in Sweden and 34% fewer cases in Stockholm County compared to the reference period in 2019, though no difference in the case fatality rate was observed [33]. Furthermore, Sweden had among the lowest pre-pandemic ICU bed capacity in Europe and was required to double it during the first wave of the pandemic [11], which may help explain the institutional responses to ED demand, including external triaging and barriers to self-referral, likely contributing to the sharp and sustained reduction in ED visits. In this study we observed that the rate of ED visits for ECSCs increased slightly between 2018 and 2019, while at the same time there was a decreasing rate of ED visits for all-causes and external causes. ECSCs require immediate and timely emergency care to improve the health outcomes of patients and an increase of ED visits for ECSC is considered indicative of more appropriate utilization [19]. Although a sharp decrease in the number of ED visits for ECSCs occurred in 2020 week 11, after the initial drop the rate of ED visits for ECSC remained stable until the end of 2021, though not returning to a similar level as observed in the years prior to the COVID-19 pandemic. Similarly, a Korean study observed a decrease in all-cause ED visits in 2020 and 2021 compared to 2019. However, this decrease was less pronounced for ECSCs, with some, such as cardiac arrest, showing an absolute increase [20]. A US-based study likewise found an increase in ED visit rates for specific ECSCs, including acute cardiovascular disease, pulmonary embolism, and pre-eclampsia, despite overall declines in all-cause ED visit rates between 2020 and 2022 [21]. Comparable patterns emerged in European settings, where the initial pandemic-related decline in ED visits was disproportionately driven by reductions in low-acuity presentations, while the proportion of patients requiring hospital admission simultaneously rose, suggesting that even patients with urgent conditions deferred care [16, 34]. Speculatively, if the reduction in ED visits was predominantly among low-acuity patients who sought care elsewhere, this may have eased staff workload and reduced overcrowding. Nevertheless, evidence indicates that some high-acuity patients also deferred and delayed seeking care during the pandemic [35]. In a Swedish cross-sectional study of patients hospitalized for acute myocardial infarction between March and June 2020, approximately one in five reported delaying care seeking due to concerns about overcrowding and the perceived risk of COVID-19 infection [36]. The finding in the present study that ED utilization had not returned to pre-pandemic levels by the end of 2021 is consistent with evidence from Italy, where overall ED volumes remained below pre-pandemic baselines through mid-2021 [37].

Despite a significant increasing trend in weekly ED visits observed across all socio-demographic groups during 2021, visit volumes did not return to pre-pandemic levels, even following the introduction of the COVID-19 vaccine in February 2021 [2]. While some of these reductions may reflect lower-acuity patients seeking alternative care, there is concern that it also signals rising unmet healthcare need, given previous studies having reported that individuals deferred or delayed seeking care during the pandemic [23–25]. This concern is essential to the interpretation of the present findings, as ECSC visit rates decreased further between 2020 and 2021, possibly indicating persistent hesitancy in seeking care. The consequences of delayed care-seeking can be serious, as a cross-country study examining subjective unmet healthcare needs among persons aged 50 and older observed a positive association between unmet need and poorer self-rated health and delayed cancer diagnosis [38]. This is especially concerning given that older people are both at higher risk of severe COVID-19 outcomes and have greater underlying healthcare needs, reflected in their more frequent use of ED care [26]. Reductions in ED utilization among this group may carry significant downstream consequences, including postponed diagnosis and treatment that could otherwise have prevented disease progression, disability, or death [14]. This is illustrated by findings from previous studies reporting that reductions in stroke-related hospitalizations during the first pandemic wave were followed by admission rates above the expected level, suggesting a rebound effect from deferred care [14, 39, 40].

Prior to the COVID-19 pandemic, ED visit rates for all-cause and external causes of morbidity decreased, potentially attributable in part to policy-driven structural changes in emergency care delivery in Stockholm. The restructuring of acute care services under the *Framtidsplanen* narrowed the scope of hospital-based general EDs, most notably at *Karolinska Sjukhuset Solna*, while the introduction of community-based urgent care centers (*närakuter*) provided care for illnesses and injuries not requiring hospital-based resources, thereby alleviating lower-acuity inflows to hospital EDs [27, 41, 42]. The further reduction in all-cause and external cause ED visits observed between 2020 and 2021 could be related to the non-pharmaceutical interventions introduced to limit viral transmission which meant that fewer people were engaging in activities that could have led to accidents and injuries such as daily commuting [2]. Moreover, ED care was reconfigured to limit the expected overcrowding including introduction of barriers to access hospital-based ED care and limit self-referring. Thus, even if a patient perceived a need for emergency care and went directly to hospital-based ED care, they could be sent home without treatment if they were assessed as low priority [27]. This strict triage is likely to have contributed to the sharp reduction of ED visits observed in this study. Notably, prior Swedish research has shown that adults aged 18–64 were more likely to self-refer to ED care and were frequently assessed as having lower medical acuity, whereas those 65 years and older were more often conveyed by ambulance, assessed as having higher medical needs, and more often admitted to inpatient care [43].

### Strengths and limitations

The use of measures that indicate specific types of ED visits such as ECSCs and external causes of morbidity, is a strength of this study as it provides insight into the reasons for attending ED care and serves as an indicator of the extent to which the ED care seeking behavior changed during the COVID-19 pandemic compared to prior years. There are several limitations that need to be considered. The study retrospectively identifies events that may have had a significant impact on the pattern of ED utilization during the pandemic. However, it is beyond the scope of this study to determine which specific regulations and recommendations drove these changes. Further, the NPR does not contain variables about contacts with primary care or the medical advice call center, restricting our ability to assess the use of alternative sources of care or inquiries regarding whether to seek emergency care. While ECSCs are based on final diagnoses recorded at the end of the patient’s ED stay, they may not fully reflect the urgency of presentations at the point of arrival, as patients presenting with symptoms warranting emergency evaluation may ultimately receive a non-serious diagnosis. Triage codes assigned at admission would provide a more direct measure of urgency independent of diagnostic outcomes. However, as triage level and codes are assigned upon arrival, they are not recorded in the NPR, precluding this comparison in the present study. Additionally, there was no possibility of directly assessing clinical severity. Furthermore, there was no information available on the number of patients who attended ED care but were sent home due to the external triaging which was set up at the beginning of the COVID-19 pandemic. Finally, as the study population was restricted to inhabitants aged 18 years and older, the full extent of the reduction in ED visits may be underestimated, given that a previous UK study found the largest reduction in ED visits was observed among schoolchildren aged 5–14 years [44].

## Conclusions

This study examined changes in the pattern of ED visits among the population of Stockholm County between 2018 and 2021, revealing that the most significant decrease in ED visits was during the COVID-19 pandemic. All socio-demographic groups followed similar trend changes with fewer ED visits, which remained lower at the end of 2021 compared to 2018–2019. The reduction in ED visits suggests that individuals may have delayed or avoided seeking necessary care, as ED visits for ECSCs did not return to pre-pandemic levels. The findings from this study highlight how populations respond to crises, and improved understanding of these changes in ED care utilization is essential for planning and adapting healthcare services to ensure timely access to care, particularly in the face of future public health emergencies. Further research is needed to understand whether the reduction in ED care seeking is due to seeking alternative sources of care or altogether foregoing necessary emergency care.

## Supplementary Information

Supplementary Material 1.

Supplementary Material 2.

## Data Availability

The datasets analysed during this current study are not publicly available due legal and ethical restrictions under the General Data Protection Regulation (GDPR), The Swedish law SFS 2018:218, The Swedish Data Protection Act, the Swedish Ethical Review Act, and the Public Access to Information and Secrecy Act. Access to these data sources may be granted to qualified researchers following legal and ethical review by the relevant Swedish Authorities. Further information regarding access procedures can be obtained from the corresponding author.

## Abbreviations

ED: Emergency Department
COVID-19: Coronavirus Disease
PPE: Personal protective equipment
NBHW: National Board of Health and Welfare
US: United States
ECSC: Emergency Care Sensitive Conditions
SEP: Socio-economic Position
RTB: Total population register
LISA: Longitudinal Integrated Database for Social Insurance and Labor Market Studies
NPR: National Patient Register
ITS: Interrupted Time Series
CI: Confidence Interval
