# Supplementary material for: The impact of the COVID-19 pandemic on emergency department visits in region Stockholm: a population-based study between 2018-2021
Source: BMC Public Health. 2026 Sep 8;26:2546. doi: 10.1186/s12889-026-29278-w (PMC13551952; doi:10.1186/s12889-026-29278-w)
Supplement: Supplementary file 1 [file 12889_2026_29278_MOESM1_ESM.docx]

**SUPPLEMENTARY MATERIAL**

**Table S1:** Description of the total number of ED visits (all-causes, Emergency care sensitive conditions (ECSC) and external causes of morbidity and injury) and relative change among the inhabitants of Stockholm County between 2018-2021.

|  | 2018 | 2019 | 2020 | 2021 | 2018-19 | 2019-20 | 2020-21 |
| --- | --- | --- | --- | --- | --- | --- | --- |
|  | No. of ED visits | | | | ▲% | ▲% | ▲% |
| *All-cause* | 413,077 | 387,559 | 315,581 | 303,991 | -6.2 | -18.6 | -3.7 |
| *ECSCs* | 47,490 | 48,136 | 35,287 | 34,857 | +2.1 | -26.7 | -1.2 |
| *Esternal causes* | 74,054 | 67,830 | 49,856 | 51,004 | -8.4 | -36.1 | +2.3 |

*▲%=relative change in the number of ED visits

**Table S2:** Description of the categories of specific causes of ED visits for external causes of morbidity and injury among the inhabitants of Stockholm County between 2018-2021.

|  | 2018 | 2019 | 2020 | 2021 |
| --- | --- | --- | --- | --- |
| *Total number of ED visits related to external causes* | 74,054 | 67,830 | 49,856 | 51,004 |
|  | **%** | **%** | **%** | **%** |
| Motor and non-motor accidents | 8.0 | 7.8 | 8.1 | 7.8 |
| Falls | 46.2 | 47.6 | 47.0 | 51.0 |
| Drowning, suffocation, burns, animals, nature, poisonings | 18.4 | 17.1 | 16.3 | 15.8 |
| Overexertion | 1.7 | 1.3 | 1.5 | 1.5 |
| Self-harm | 2.2 | 2.6 | 3.1 | 2.1 |
| Assualt | 5.6 | 5.0 | 5.5 | 4.7 |
| Complications due to medication or ongoing treatment | 6.2 | 6.1 | 6.1 | 6.9 |
| Unspecified | 11.9 | 12.4 | 12.1 | 10.1 |

*% the proportion of ED visi

**Table S3:** The socio-demographic differences the rate of ED visits for ECSCs and rate of EDv visits related to the external causes of morbidity per 1,000 persons between 2018-2021.

|  | **Age adjusted rate of visits per 1,000 persons** | | | | | | | |
| --- | --- | --- | --- | --- | --- | --- | --- | --- |
|  | **ED visits for ECSC** | | | | **ED visits due to external causes** | | | |
|  | **2018** | **2019** | **2020** | **2021** | **2018** | **2019** | **2020** | **2021** |
|  | 22.6 | 23.0 | 16.8 | 16.6 | 65.5 | 60.6 | 44.7 | 46.2 |
| ***Sex*** |  |  |  |  |  |  |  |  |
| female | 11.7 | 11.9 | 8.6 | 8.5 | 34.3 | 31.8 | 23.3 | 24.5 |
| Male | 10.9 | 11.0 | 8.2 | 8.1 | 31.2 | 28.8 | 21.4 | 21.7 |
| ***Age groups*** |  |  |  |  |  |  |  |  |
| 18-39yrs | 3.6 | 3.4 | 2.4 | 2.3 | 17.2 | 15.0 | 10.7 | 10.1 |
| 40-49yrs | 2.1 | 1.9 | 1.5 | 1.4 | 8.2 | 7.4 | 5.3 | 5.7 |
| 50-59yrs | 2.8 | 2.8 | 2.1 | 2.2 | 9.5 | 8.4 | 6.2 | 6.7 |
| 60-69yrs | 4.0 | 3.9 | 2.9 | 3.0 | 9.3 | 8.3 | 5.9 | 6.4 |
| 70-79yrs | 5.0 | 5.3 | 3.8 | 4.1 | 9.7 | 9.2 | 6.9 | 8.1 |
| 80+yrs | 5.1 | 5.7 | 4.1 | 3.8 | 11.7 | 12.2 | 9.5 | 9.3 |
| ***Country of birth*** |  |  |  |  |  |  |  |  |
| Other | 5.9 | 5.9 | 4.4 | 4.3 | 16.6 | 15.0 | 10.8 | 11.5 |
| Sweden | 16.7 | 17.1 | 12.5 | 12.3 | 48.9 | 45.6 | 33.9 | 34.7 |
| ***Education level*** |  |  |  |  |  |  |  |  |
| Primary | 5.3 | 5.4 | 4.0 | 3.9 | 13.9 | 12.7 | 9.6 | 9.7 |
| Secondary | 10.3 | 10.6 | 7.8 | 7.6 | 30.8 | 28.5 | 20.9 | 21.6 |
| Tertiary | 6.4 | 6.3 | 4.7 | 4.8 | 19.3 | 17.9 | 13.1 | 13.9 |
| Missing | 0.5 | 0.6 | 0.4 | 0.4 | 1.5 | 1.4 | 1.0 | 1.1 |
| ***Income groups*** |  |  |  |  |  |  |  |  |
| (lowest) group 1 | 6.7 | 6.8 | 5.0 | 4.7 | 17.4 | 16.5 | 12.7 | 12.4 |
| group 2 | 5.7 | 6.0 | 4.3 | 4.4 | 15.3 | 14.7 | 10.9 | 11.3 |
| group 3 | 3.6 | 3.7 | 2.7 | 2.7 | 11.5 | 10.3 | 7.5 | 8.0 |
| group 4 | 3.2 | 3.1 | 2.3 | 2.4 | 10.5 | 9.2 | 6.6 | 7.0 |
| (highest(group 5 | 3.2 | 3.2 | 2.3 | 2.4 | 10.4 | 9.3 | 6.7 | 7.1 |
| Missing | 0.1 | 0.1 | 0.1 | 0.1 | 0.4 | 0.5 | 0.3 | 0.4 |

**Figure S1:** The trends in the weekly number of ED visits among all inhabitants of Stockholm County between 2018 and 2021. The hollow navy circles represent the weekly number of ED visits, the vertical dotted line represents 2020 week 11 when high risk of community spread was declared, and the solid red lines present the trends in ED visits.

**Figure S2:** The trends in the weekly number of ED visits stratified by socio-demographic groups in Stockholm County between 2018 and 2021. The hollow navy circles represent the weekly number of ED visits, the vertical dotted line represents 2020 week 11 when high risk of community spread was declared, and the solid red lines present the trends in ED visits. A) Inhabitants 70 years and older B) Male inhabitants. C) Female inhabitants. D) Inhabitants born outside of Sweden. E) Primary educated. F) Lowest income group.
